# Utilization of Split-Thickness Skin Graft as a Treatment Option Following Mohs Micrographic Surgery

**DOI:** 10.7759/cureus.53652

**Published:** 2024-02-05

**Authors:** Luis J Borda, Courtny S Cushman, Thomas W Chu

**Affiliations:** 1 Department of Dermatology, Eastern Virginia Medical School, Norfolk, USA

**Keywords:** pinch graft, hard-to-heal wound, lower leg, mohs micrographic surgery, split thickness skin graft

## Abstract

Split-thickness skin grafting (STSG) is a frontline treatment for challenging surgical wounds, including diabetic foot ulcers, venous leg ulcers, and post-surgical defects. This study explores the use of STSG employing the pinch graft technique for hard-to-heal surgical wounds following Mohs micrographic surgery (MMS). An 83-year-old patient with a non-improving post-MMS defect on the left lower leg underwent STSG from the right inner thigh using the pinch graft technique. The grafts were secured with a mesh dressing, adhesive strips, and compression bandaging. The patient experienced complete re-epithelialization and reduced pain within five weeks, emphasizing the efficacy of STSG for challenging cases. This case underscores the importance of considering STSG, especially in challenging locations, as a rapid and efficient treatment with improved quality of life. The pinch graft technique is presented as a useful option following MMS. This study encourages Mohs surgeons to consider STSG for reconstruction in challenging locations, especially on the lower leg.

## Introduction

Split-thickness skin grafting (STSG) is one of the first-line treatment options for diabetic foot ulcers, venous leg ulcers, and post-surgical defects of the lower leg that cannot be closed primarily or where undue tension is predicted [1]. It can therefore present as an alternative option for the reconstruction of hard-to-close Mohs micrographic surgery (MMS) defects. In the pinch graft technique, the donor graft can be harvested from the ipsilateral thigh, arm's volar surface, or abdomen using local anesthesia, enabling effective primary closure. After anesthesia, the harvest site is aseptically scrubbed and prepped, with marking off the area recommended. Tissue quantity depends on the recipient site size. A shave-biopsy blade is used for harvesting, ensuring the blade handle remains parallel during pinch graft retrieval in order to prevent cutting through the dermis into the fat layer. Once all the pinch grafts are on the recipient wound bed, the recipient site can be covered with a non-adherent dressing [1,2]. Alternatively, compression bandaging can be placed (especially if the ulcer is located on the lower leg) to accelerate wound healing. We present the use of STSG utilizing the pinch graft technique [2,3] as an option for hard-to-heal surgical wounds following MMS.

## Case presentation

**Figure 1:**
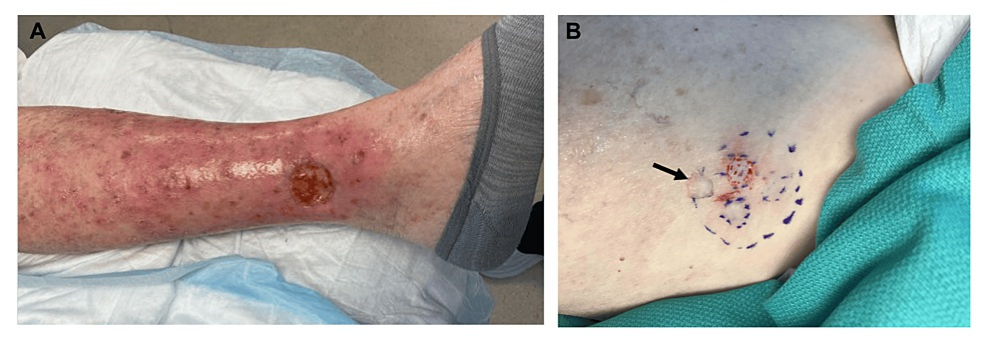
Recipient and donor site before split-thickness skin grafting. (A) Surgical wound (recipient site) with yellow wound bed with raised borders on the left lower leg, postoperative day 90; (B) Right inner thigh donor site with one graft harvested (arrow)

An 83-year-old female with a history of hypertension presented for the follow-up of a hard-to-heal surgical wound after MMS of melanoma in situ on the left lower leg. The 3x3 cm surgical defect above the left medial malleolus was initially left to heal by secondary intention. After three months, the site had no significant improvement. Physical exam showed a round ulcer with raised borders and yellow exudate on the wound bed. There was mild erythema on the surrounding skin due to constant dressing changes (Figure 1A). No purulent drainage, foul odor, or inguinal or popliteal lymphadenopathies were observed. The patient denied any fever or chills. She also described experiencing persistent throbbing pain in the vicinity of the ulcer and reported associated discomfort during ambulation.

STSGs in the form of four pinch grafts were harvested from the skin at the right inner thigh (Figure 1B). Light debridement of the recipient site was performed before graft placement.

**Figure 2:**
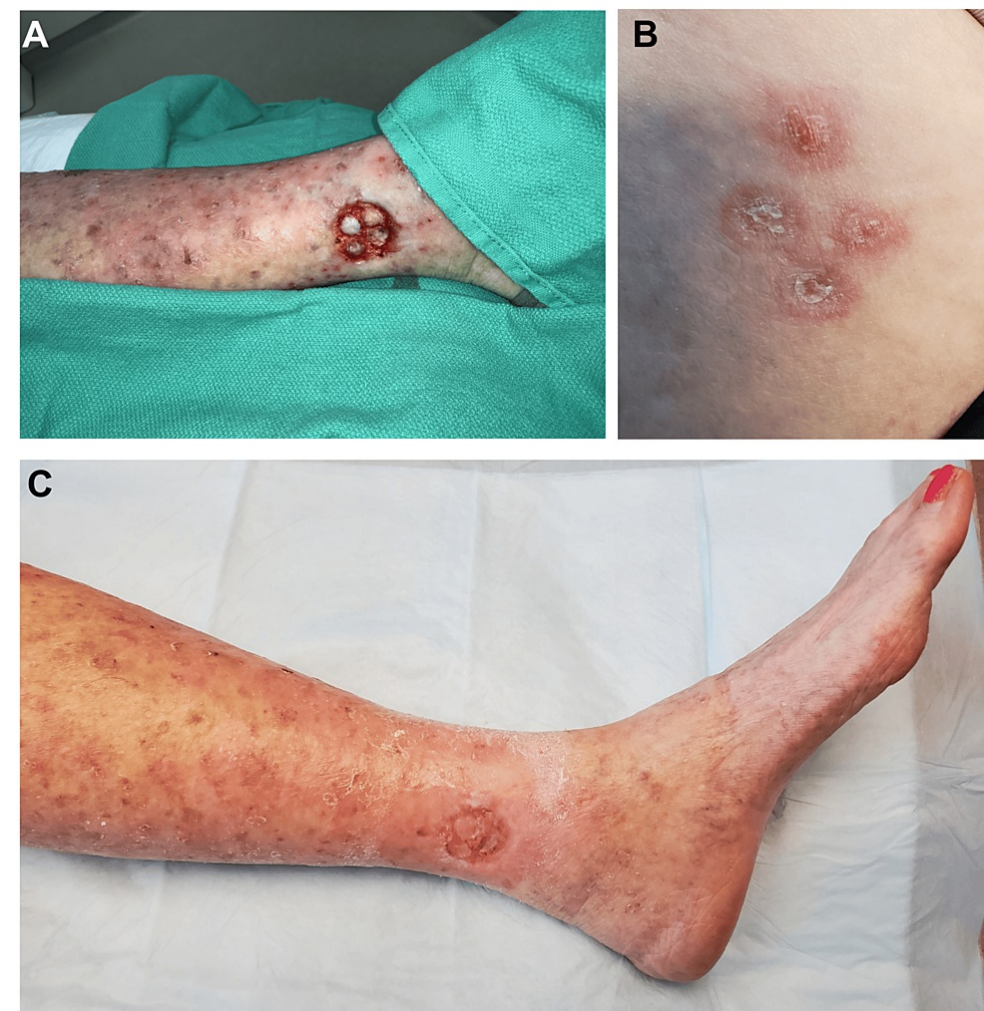
Recipient and donor site after split-thickness skin grafting (STSG) (A) Surgical wound (recipient site) with harvested STSGs on wound bed; (B) Donor site at five weeks post grafting; (C) Recipient site completely epithelialized at five weeks post grafting

The grafts were secured in place by petroleum jelly-impregnated mesh dressing, adhesive strips, and compression bandaging. Non-adherent pad and film dressing were applied to the donor site post procedure. Furthermore, the patient received a two-week course of cephalexin 500 mg twice daily. Complete healing with minimal scarring at the right inner thigh donor site was achieved by the second week. Weekly dressing changes were performed with complete re-epithelialization of the lower leg wound five weeks later (Figure 2). The patient also noted a reduction in pain while walking, resulting in the ability to engage in more activities of daily living.

## Discussion

Traditionally, leg ulcers deemed chronic are those that do not exhibit healing within a three-month period or fail to demonstrate a reduction in total surface area ranging from 20% to 40% after two to four weeks of optimal therapeutic intervention [3], as seen in our case.

A STSG is defined as a graft that comprises the epidermis and a portion of the dermis. Unlike flaps, skin grafts do not possess an autonomous blood supply; instead, they depend on a well-vascularized wound bed for graft survival. STSGs are accessible from diverse anatomical locations and in varying thicknesses. Typically, STSG autografts are harvested from the lateral thigh and trunk, selected for both their aesthetic concealment and the ease of harvest facilitated by their broad surfaces [4]. The perimeter and the central portion of STSGs need to be secured to the wound bed. Basting sutures or any pressure dressing (as shown in our case) may be placed centrally ensuring good donor and recipient tissue apposition [5].

In comparison to full-thickness skin grafts, STSGs exhibit higher survival rates. These thin grafts, requiring less tissue revascularization, are well-suited for areas with limited vascular supply. STSGs can be directly applied over the periosteum, perichondrium, perineurium, and peritenon, while full-thickness skin grafts should be avoided in these recipient sites. STSGs present greater contraction (caution needed around joints) and inferior cosmesis compared to full-thickness skin grafts (Figure 2B) [6]. Anticipated outcomes include color and textural mismatch due to the absence of adnexal structures [7], since STSGs are often sourced from sites lacking similar vascular patterns, sebaceous qualities, thickness, texture, and sun exposure. The absence of adnexal structures leads to scaling, crusting, and resulting xerosis. Prior reports indicate the potential of using pinch grafting for the management of various chronic leg ulcers. Some authors have proposed pinch grafting as an adjunct to conservative wound care and as a primary transplantation method [8]. It is a simple, secure, and cost-effective procedure that demands minimal resources.

Surgical wounds may represent a challenge to healing, especially on the lower extremities [9]. STSG offers advantages for optimal patient outcomes by allowing significant surface area coverage with smaller donor sites that heal without the need for closure and can even be re-harvested [1]. In addition, the advantages of STSG become particularly evident in surgical defects that are hard to close primarily, as exemplified by our case post-MMS on the lower extremity in a patient with comorbidities. Furthermore, skin grafts, particularly STSG, may aid in the early detection of tumor recurrence in cutaneous oncology [5]. Therefore, as demonstrated in our case, STSG is not only a rapid and efficient treatment option, but it also provides patients with a better health-related quality of life compared with second-intention healing, especially when coupled with compression therapy.

Finally, following MMS, there may be postoperative regional lymphadenopathy, resulting from an inflammatory reaction. Typically, this occurs several days after the surgery and manifests as lymph nodes measuring less than 1.0 cm in size [10,11]. Mohs surgeons, dermatologists, and other healthcare providers should exert caution not to confuse this reactive lymphadenopathy as potential lymph node metastasis.

## Conclusions

This study highlights the effectiveness of STSG using the pinch graft technique as a valuable option for managing hard-to-heal surgical wounds, specifically in the context of MMS on the lower extremities. The presented case of an 83-year-old patient with a non-healing surgical wound post-MMS demonstrates successful outcomes following STSG, showcasing complete healing. The advantages of STSG, including its adaptability to challenging wound locations, efficient coverage, and potential for re-harvesting, make it a favorable option for optimal patient outcomes. Additionally, STSG's role in early tumor recurrence detection in cutaneous oncology adds to its clinical significance. This study emphasizes the benefits of STSG over second-intention healing, especially when coupled with compression therapy, and underscores the importance of recognizing postoperative regional lymphadenopathy as a non-metastatic inflammatory response following MMS (although not seen in our case). Overall, these findings support STSG as an efficient treatment option for hard-to-close surgical wounds.
